# Protective Effect of a Cocoa-Enriched Diet on Oxidative Stress Induced by Intensive Acute Exercise in Rats

**DOI:** 10.3390/antiox11040753

**Published:** 2022-04-10

**Authors:** Patricia Ruiz-Iglesias, Malén Massot-Cladera, Maria J. Rodríguez-Lagunas, Àngels Franch, Mariona Camps-Bossacoma, Francisco J. Pérez-Cano, Margarida Castell

**Affiliations:** 1 Secció de Fisiologia, Departament de Bioquímica i Fisiologia, Facultat de Farmàcia i Ciències de l’Alimentació, Universitat de Barcelona (UB), 08028 Barcelona, Spain; patriciaruiz@ub.edu (P.R.-I.); malen.massot@ub.edu (M.M.-C.); mjrodriguez@ub.edu (M.J.R.-L.); angelsfranch@ub.edu (À.F.); marionacamps@ub.edu (M.C.-B.); 2 Institut de Recerca en Nutrició i Seguretat Alimentària (INSA-UB), 08921 Santa Coloma de Gramenet, Spain; 3 Centro de Investigación Biomédica en Red de Fisiopatología de la Obesidad y la Nutrición (CIBEROBN), Instituto de Salud Carlos III, 28029 Madrid, Spain

**Keywords:** acute exercise, fiber, flavanols, immunoglobulin, leukocytes, lymphocytes, oxidative stress, polyphenols, ROS

## Abstract

Intensive acute exercise can induce oxidative stress, leading to muscle damage and immune function impairment. Cocoa diet could prevent this oxidative stress and its consequences on immunity. Our aim was to assess the effect of a cocoa-enriched diet on the reactive oxygen species (ROS) production by peritoneal macrophages, blood immunoglobulin (Ig) levels, leukocyte counts, and the physical performance of rats submitted to an intensive acute exercise, as well as to elucidate the involvement of cocoa fiber in such effects. For this purpose, Wistar rats were fed either a standard diet, i.e., a diet containing 10% cocoa (C10), or a diet containing 5% cocoa fiber (CF) for 25 days. Then, half of the rats of each diet ran on a treadmill until exhaustion, and 16 h later, the samples were obtained. Both C10 and CF diets significantly prevented the increase in ROS production. However, neither the cocoa diet or the cocoa fiber-enriched diet prevented the decrease in serum IgG induced by acute exercise. Therefore, although the cocoa-enriched diet was able to prevent the excessive oxidative stress induced by intensive exercise, this was not enough to avoid the immune function impairment due to exercise.

## 1. Introduction

Cocoa beans are the seeds of the fruit from the *Theobroma cacao* L. tree. After fermentation, drying, and subsequent processing, cocoa powder is obtained. Over the last centuries, some medicinal properties have been attributed to cocoa [1], but it has only been in recent years that cocoa has become a subject of increasing interest because of its several beneficial effects on human health, including as an antioxidant [2], a blood pressure regulator [3] and its immunomodulatory [4] and anti-inflammatory [5] properties. These effects are mainly attributed to its polyphenol content, which mostly consists of flavanols and their polymers. More specifically, cocoa polyphenol content comprises about 58% of proanthocyanidins, 37% of catechins, and 4% of anthocyanidins [6]. The predominant catechin monomer found in cocoa is (–)-epicatechin [6], and given its high abundance, it is believed that it could play an essential role in the beneficial effects of cocoa. For this reason, several authors have focused their attention on the isolated effect of this catechin on exercise performance [7], muscle oxygenation [8], and cytokine production [9], among others [10]. However, it is important to consider that cocoa also contains other bioactive compounds, such as dietary fiber and methylxanthines, which may contribute to cocoa’s beneficial effects on health and particularly on the immune system [11,12].

Acute intensive exercise often triggers an imbalance between endogenous oxidants and antioxidants, in favor of the oxidants, a phenomenon that is commonly known as oxidative stress [13]. Reactive oxygen species (ROS) are oxygen-containing reactive molecules and free radicals generated as a result of molecular oxygen reduction during normal cellular metabolism processes [14]. They are involved in many physiological processes, such as protein phosphorylation, transcriptional factors activation, immune system signaling, and apoptosis [14]. However, an excessive accumulation of extracellular free radicals impairs the immune system functionality, leading to a systemic inflammatory status [15] that could contribute to the development of a great number of pathologies, such as autoimmune, metabolic and neurodegenerative diseases, or even cancer [16]. In the athletic area, muscle damage, physical fatigue, and an impaired exercise performance have also been associated with an overproduction of ROS induced by intensive exercise [17]. For this reason, in this field, supplementation with exogenous antioxidants to counteract these phenomena and, consequently, improve exercise performance [17] is of increasing interest. Moreover, the prescription of exercise-based rehabilitation programs in the clinical practice has grown lately. The mentioned programs are considered a safe intervention [18] to enhance endogenous antioxidant systems through sirtuin 1 (SIRT1) activation [19], among other beneficial effects; however, exogenous antioxidant supplementation may be a useful complementary therapy in patients with diseases associated with oxidative stress [20]. 

Exogenous antioxidants can be obtained through most fruits and vegetables, but the amount of them present in a standard diet may be insufficient to support intensive exercise demands. Hence, some commercial supplements containing polyphenols and/or other molecules with antioxidant properties, such as vitamins E, C, and A and some minerals, are available [17]. Polyphenols exert their antioxidant capacity mainly through the direct neutralization of free radicals and chelating metals such as Fe^2+^ and Cu^+^ [21]. However, other relevant mechanisms have been reported, such as the stimulation of mitochondrial biogenesis through the activation of SIRT1 [19,22] and nuclear factor (erythroid-derived 2)-like 2 (Nrf2) [23] signaling pathways. Cocoa, due to its polyphenol content, seems a good source of exogenous antioxidants that may be useful for preventing some of the alterations induced by intensive exercise, such as muscular damage [24], immune disruption [25,26,27], and even the decline in performance, although the available evidence does not support the use of cocoa as an ergogenic aid [28]. Moreover, isolated cocoa fiber, even containing a much lower percentage of polyphenols, has also demonstrated antioxidant properties [29,30], which, together with its beneficial effects in the intestinal compartment [31], may also be a good supplement to avoid the undesirable effects of overly intensive exercise [32].

We previously demonstrated that hesperidin supplementation, which is the main polyphenol found in citrus fruits, was able to prevent the overproduction of ROS induced by exhausting exercise through enhancing the endogenous antioxidant systems of intensively trained rats [33]. Moreover, we recently found the beneficial effects of cocoa, especially from its fiber content, on the cecal microbiota and the mucosal immune system of rats submitted to acute exercise [34]. Here, we aimed to evaluate the effect of a cocoa-enriched diet on the physical performance, the ROS production by peritoneal macrophages, and the systemic immune function of rats submitted to intensive acute exercise, as well as to elucidate the involvement of cocoa fiber in such effects.

## 2. Materials and Methods

### 2.1. Animals

Female Wistar rats (four-week-old) were provided by Envigo (Huntingdon, UK) and maintained in the animal facilities of the Faculty of Pharmacy and Food Science at the University of Barcelona. Female rats were used, because they showed a better adaptation to the treadmill and exercise performance than male rats in previous studies from our laboratory [25] and from others [35], whereas the impact of exercise on immunological variables was not influenced by rat gender [25]. The rats were kept in polycarbonate cages, with four animals per cage, under controlled conditions of temperature and humidity in a 12 h light/12 h dark cycle and had ad libitum access to food and water. The animal procedure was approved by the Ethical Committee for Animal Experimentation of the University of Barcelona and the Catalonia Government (CEEA/UB ref. 517/18 and DAAM 9257, respectively). All methods were carried out in accordance with relevant guidelines and regulations.

### 2.2. Nutritional and Exercise Intervention

The nutritional and exercise intervention applied in the current study has already been reported [34]. Briefly, all rats were first familiarized with running on a rodent treadmill (Exer3/6, Columbus, OH, USA) for one week, with the increasing running time and speed. Then, animals performed an exhaustion test (ET) in which, after 10 min running at 18 m/min, the speed was increased every 2 min (by 3 m/min) until rat exhaustion. The end of the test was established, when rats touched the shock grid more than three times. Rats were then homogeneously distributed in six groups according to their running capacity (n = 8/each)—REF/C, REF/R, C10/C, C10/R, CF/C, and CF/R—that received three different isoenergetic diets during 25 days as in previous studies [31]. The R groups would run again after the 25-day diet, whereas the C groups would not.

The reference (REF) groups (i.e., REF/C and REF/R groups) received a standard diet AIN-93M (maintenance diet from the American Institute of Nutrition, Envigo, Huntingdon, UK). The cocoa (C10) groups (i.e., C10/C and C10/R groups) were fed a diet containing 10% defatted cocoa (Idilia Foods S.L., Barcelona, Spain) providing a final proportion of 3.6 g/kg polyphenols, 6.0 g/kg soluble fiber, and 54.0 g/kg insoluble fiber. The cocoa fiber (CF) groups (i.e., CF/C and CF/R groups) were fed a diet containing 5% cocoa fiber powder (Idilia Foods S.L.), which provided 0.4 g/kg of polyphenols, 8.0 g/kg soluble fiber, and 56.0 g/kg insoluble fiber. Thus, the CF diet provided a similar proportion of fiber to the C10 diet but much lower amount of polyphenols, as applied in previous studies [11]. 

One week prior to the end of the dietary intervention, all rats were again familiarized with running on the treadmill. The final ET, only performed by the R animals, consisted of 15 min running at 18 m/min and then every 2 min with a speed increased by 3 m/min until exhaustion. This ET was carried out in the afternoon, between 5 and 8 p.m. 

### 2.3. Sample Collection

Sixteen hours after the ET of R rats, both R and C rats were euthanized. For this, animals were anesthetized intramuscularly with ketamine (90 mg/kg; Merial Laboratories S.A. Barcelona, Spain) and xylazine (10 mg/kg; Bayer A.G., Leverkusen, Germany). Peritoneal macrophages were immediately collected and used to assess ROS production. Moreover, anticoagulated blood (EDTA-K_3_) was obtained from the heart and was immediately analyzed using an automated hematology analyzer (Spincell, MonLab Laboratories, Barcelona, Spain). Other blood samples were used to obtain plasma and serum, which were maintained at −80 or −20 °C until hormone and immunoglobulin (Ig) quantification, respectively. The spleen and heart were also collected and weighed.

### 2.4. Peritoneal Macrophages Isolation and ROS Production 

To collect the peritoneal macrophages, 40 mL of cold, sterile phosphate-buffered saline (PBS, pH 7.2) were injected into the peritoneal cavity for 2 min under massage and immediately collected, as described previously [33,36]. The cell suspension obtained was centrifuged (speed: 538× *g*; duration: 10 min; temperature: 4 °C) and resuspended with cold Roswell Park Memorial Institute (RPMI) media without phenol red, supplemented with 10% heat-inactivated fetal bovine serum, 100 IU/mL streptomycin–penicillin, and 2 mM L-glutamine (all from Sigma-Aldrich, Madrid, Spain). After counting the macrophages using a Spincell hematology analyzer (MonLab Laboratories), 10^4^ cells/well were plated on a black 96-well plate (Thermo Fisher Scientific, Barcelona, Spain) and incubated overnight to allow their attachment to the plate. On the next day, cells were washed with warm RPMI media, and the ROS production was assessed after incubation for 30 min with 20 µM of reduced 2′,7′-dichlorofluorescein diacetate probe (H2DCF-DA; Invitrogen, Paisley, UK) in order to oxidize the H2DCF-DA to a fluorescent compound (2′,7′-dichlorofluorescein) [33,36]. Then, 0.5 mM of H_2_O_2_ was added to the plate, and the fluorescence was measured once every 15 min for 2 h by the fluorimeter Modulus Microplate Multimode Reader (Turner BioSystems, Sunnyvale, CA, USA). 

### 2.5. Plasma Cortisol and Noradrenaline (NA) Concentration

The quantification of cortisol in plasma was performed with the DetectX^®^ Cortisol competitive enzyme-linked immunosorbent assay (ELISA; Arbor Assays, Ann. Arbor, MI, USA) following the manufacturer’s instructions. Plasma NA concentration was quantified using an NA/norepinephrine (NA/NE) competitive ELISA Kit (Elabscience, Houston, TX, USA) following the manufacturer’s instructions. In both cases, absorbance was measured on a microplate photometer (Labsystems Multiskan), and data were interpolated by Ascent v.2.6 software (Thermo Fisher Scientific) according to the respective standard.

### 2.6. Serum Immunoglobulins Concentration

The concentrations of IgG, IgM, and IgA in sera were determined by a sandwich ELISA (Bethyl Laboratories Inc., Montgomery, TX, USA), as previously detailed [37]. Results were analyzed in a microplate photometer, as mentioned before, and data were interpolated by the same software.

### 2.7. Statistical Analysis

The analysis of the data was performed using IBM Social Sciences Software Program (SPSS, version 26.0; Chicago, IL, USA). Once the normality and equality of the data were confirmed by a Shapiro–Wilk and Levene test, respectively, a two-way ANOVA test was applied. If significant differences were detected, Tukey’s post hoc test was carried out. When the data were neither equally nor normally distributed, a Kruskal–Wallis test followed by a Mann–Whitney U test was applied. Significant differences were considered for *p* < 0.05.

## 3. Results

### 3.1. Exercise Performance

**Figure 1:**
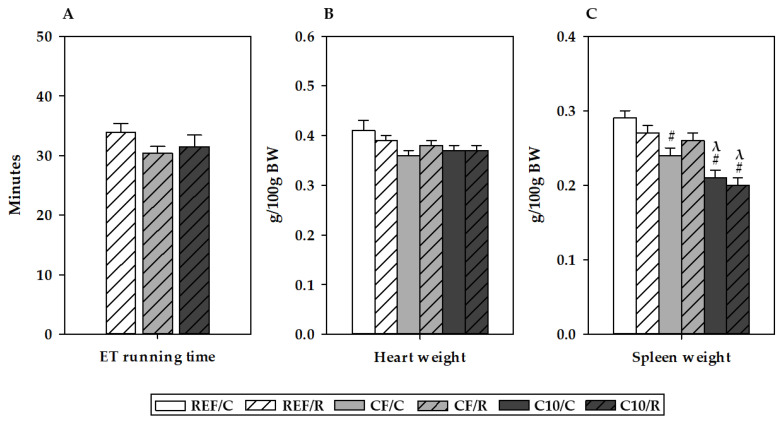
Running time during the final exhaustion test for running (R) rats (**A**) and relative heart (**B**) and spleen (**C**) weights for all groups. REF, reference diet; CF, 5% cocoa fiber-enriched diet; C10, 10% cocoa-enriched diet. The control (C) groups are represented by smooth bars, and the R groups are represented by striped bars. Data are expressed as mean ± standard error of the mean (SEM) (n = 8). Statistical differences (a Kruskal–Wallis test followed by a Mann–Whitney U test): ^#^ *p* < 0.05 vs. the same exercise condition in the REF diet; ^λ^ *p* < 0.05 vs. the same exercise condition in the CF diet.

The time lasted in the final ET (Figure 1a) was monitored as a measure of exercise performance. This time was about 30 min in control rats, and the diets enriched in cocoa or cocoa fiber did not statistically modify it. 

### 3.2. Organ Weight and Plasma Cortisol and NA Concentrations

Sixteen hours after performing the final ET, tissue and blood samples were obtained. Acute exercise did not modify the weight of the heart or spleen (Figure 1b,c). Likewise, the heart weight was not influenced by the C10 or CF diets. However, the intake of the C10 diet lowered the relative spleen weight in C10/C and C10/R animals (*p* = 0.001), whereas the CF diet decreased it in sedentary CF/C rats (*p* = 0.007).

**Figure 2:**
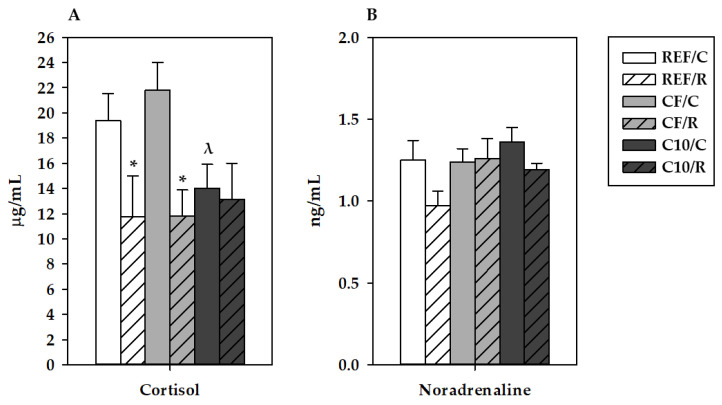
Plasma cortisol (**A**) and noradrenaline (**B**) concentrations 16 h after performing the final exhaustion test. REF, reference diet; CF, 5% cocoa fiber-enriched diet; C10, 10% cocoa-enriched diet. The control (C) groups are represented by smooth bars, and the runner (R) groups are represented by striped bars. Data are expressed as mean ± SEM (n = 8). Statistical differences (a Kruskal–Wallis test followed by a Mann–Whitney U test): * *p* < 0.05 vs. the C group in the same diet; ^λ^ *p* < 0.05 vs. the same exercise condition in the CF diet.

On the other hand, 16 h after the final ET, REF/R rats showed a lower concentration of plasma cortisol than REF/C animals (about a 40% decrease, *p* = 0.035; Figure 2a). The C10 diet decreased the plasma cortisol concentration in C10/C animals, with this change only being significant when it was compared with in the CF/C group (*p* = 0.016). Neither the acute exercise nor the experimental diets induced statistically significant changes in plasma NA (Figure 2b).

### 3.3. Hemograme

**Figure 3:**
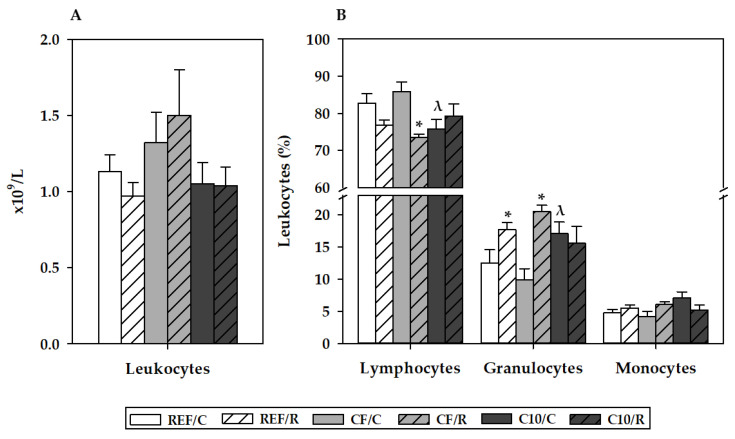
Blood counts of leukocytes (**A**) and proportions of lymphocytes, granulocytes, and monocytes (**B**) 16 h after performing the final exhaustion test. REF, reference diet; CF, 5% cocoa fiber-enriched diet; C10, 10% cocoa-enriched diet. The control (C) groups are represented by smooth bars, and the runner (R) groups are represented by striped bars. Data are expressed as mean ± SEM (n = 8). Statistical differences (a Kruskal–Wallis test followed by a Mann–Whitney U test): * *p* < 0.05 vs. the C group in the same diet; ^λ^ *p* < 0.05 vs. the same exercise condition in the CF diet.

Blood leukocyte counts were similar in animals fed standard diet and in animals fed CF and C10 diets after 16 h of a single bout of intensive exercise (Figure 3a). With regard to differential leukocytes (Figure 3b), acute exercise in animals following REF and CF diets, but not C10 diet, induced a decrease in the proportion of lymphocytes, which achieved statistical significance only for the CF group (*p* = 0.009, CF/C vs. CF/R; *p* = 0.07, REF/C vs. REF/R). Reciprocally, there was an increase in the percentage of granulocytes by acute exercise in animals fed REF and CF diets (*p* = 0.009, CF/C vs. CF/R; *p* = 0.05, REF/C vs. REF/R). The percentage of monocytes was not modified either by acute exercise or by the intake of the experimental diets. 

**Figure 4:**
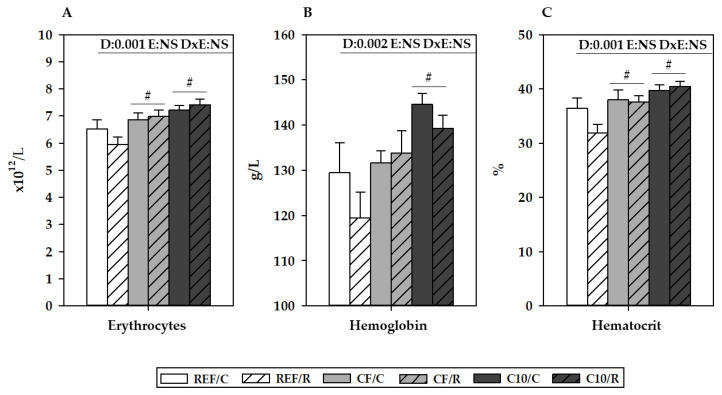
Blood erythrocyte counts (**A**), hemoglobin concentration (**B**), and hematocrit (**C**) 16 h after performing the final exhaustion test. REF, reference diet; CF, 5% cocoa fiber-enriched diet; C10, 10% cocoa-enriched diet. The control (C) groups are represented by smooth bars, and the runner (R) groups are represented by striped bars. Data are expressed as mean ± SEM (n = 8). Statistical differences (two-way ANOVA followed by Tukey’s post-hoc test): ^#^ *p* < 0.05 vs. the REF diet.

With regard to results related to red blood cells (Figure 4), erythrocyte counts, hemoglobin (HGB) concentration, and hematocrit (HCT) were not affected after 16 h of an acute exercise. However, there was an increase in the erythrocyte counts and HCT after both C10 and CF diets (*p* < 0.05) and in the HGB concentration after following the C10 diet (*p* = 0.001). 

### 3.4. ROS Production by Peritoneal Macrophages

**Figure 5:**
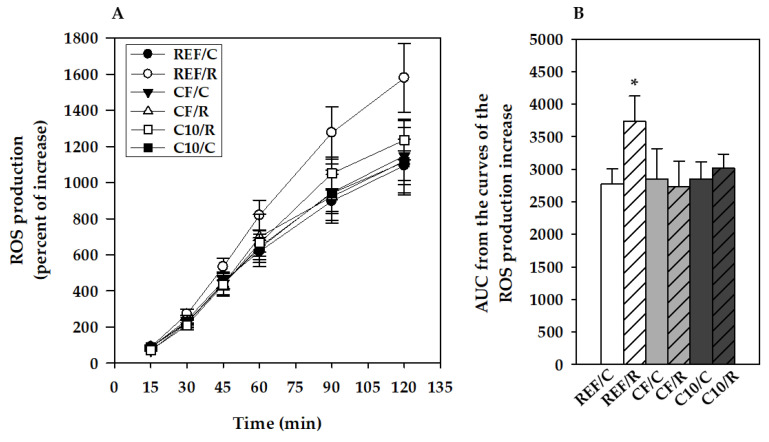
Reactive oxygen species (ROS) production by peritoneal macrophages 16 h after performing the final exhausion test. (**A**) Kynetics of ROS production for 2 h; (**B**) Area under the curve (AUC) between 15–120 min. REF, reference diet; CF, 5% cocoa fiber-enriched diet; C10, 10% cocoa-enriched diet; C, control groups; R, runner groups. Data are expressed as mean ± SEM (n = 8). Statistical differences (a Kruskal–Wallis test followed by a Mann–Whitney U test): * *p* < 0.05 vs. the C group in the same diet.

The ROS production by peritoneal macrophages in runner rats fed REF diet was higher than that of REF/C rats (*p* = 0.037) (Figure 5). This exercise-induced increase was prevented by both experimental diets.

### 3.5. Serum Immunoglobulins

**Figure 6:**
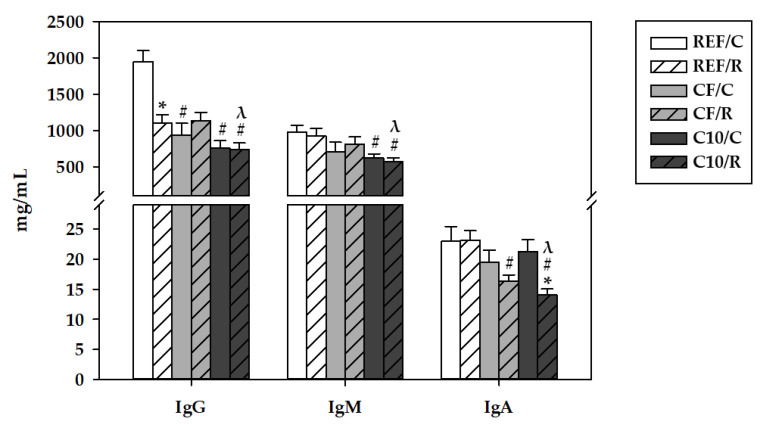
Serum concentrations of IgG, IgM and IgA 16 h after performing the final exhaustion test. REF, reference diet; CF, 5% cocoa fiber-enriched diet; C10, 10% cocoa-enriched diet. The control (C) groups are represented by smooth bars, and the runner (R) groups are represented by striped bars. Data are expressed as mean ± SEM (n = 8). Statistical differences (a Kruskal–Wallis test followed by a Mann–Whitney U test): * *p* < 0.05 vs. the C group in the same diet; ^#^ *p* < 0.05 vs. the same exercise condition in the REF diet; ^λ^ *p* < 0.05 vs. the same exercise condition in the CF diet.

The serum concentrations of IgG, IgM, and IgA were determined in samples obtained 16 h after the final ET (Figure 6). Acute exercise induced a 50% decrease in IgG (*p* = 0.003), whereas it did not affect the levels of IgM and IgA.

With regard to the nutritional intervention, both C10 and CF diets lowered the serum IgG levels in the sedentary C groups (*p* < 0.005), and they were not influenced by exercise. The C10 diet also induced a decrease in serum IgM, independently of the exercise conditions (*p* < 0.05). On the other hand, acute exercise decreased the serum concentrations of IgA in rats fed C10 and CF diets (*p* < 0.005 vs. REF/R).

## 4. Discussion

Previous preclinical studies have reported the antioxidant, immunomodulatory, and anti-inflammatory effects of a cocoa-enriched diet in preclinical models of immune-mediated diseases such as arthritis [36,38], allergy [39,40], and inflammation [41]. Moreover, dietary interventions with cocoa fiber have shown beneficial effects, not only in the intestinal compartment [31], but also in the oxidative stress induced by an inflammatory state, such as metabolic syndrome [29]. However, to our knowledge, there are no preclinical studies focused on the beneficial properties of cocoa on the oxidative stress induced by intensive exercise. Moreover, clinical studies involving cocoa interventions are scarce, and they are mainly focused on cocoa’s effects on endothelial function and cardiovascular health [42]. Focusing on the exercise field, there are some clinical studies [2,43,44] and some reviews [28,45,46] that assess the ergogenic and antioxidant potential of cocoa and its polyphenols in athletes; however, the results are not clear enough to draw any solid conclusions. In addition, most of the studies assessed the effects of the acute intake of cocoa without focusing on the long-term effects and do not evaluate the impact on immune function. In the current study, we have studied the potential protective effect of a dietary intervention with cocoa on the overproduction of ROS and the consequent immune function impairment induced by intensive running exercise in rats. We have also approached the effect of a dietary intervention with isolated cocoa fiber in these variables, which provides far fewer polyphenols but, in previous studies, has also demonstrated antioxidant and immunomodulating properties [29,31]. 

Here, both cocoa- and cocoa fiber-enriched diets prevented the increase in ROS production by peritoneal macrophages induced by acute exercise. The preventive effect of the diets may be attributed to the scavenging activity of polyphenols, since due to their chemical structure they can directly neutralize free radicals such as superoxide anions, hydroxyl, peroxynitrite, and nitric oxide radicals by transferring their own electrons [47]. Other mechanisms may be involved, since other polyphenols such as the flavonol quercetin have been shown to stimulate mitochondrial biogenesis throughout enhancing the expression of SIRT1 and PGC-1α, as well as increasing the cytochrome C concentrations in the muscles and brain [22]. Moreover, resveratrol, a non-flavonoid polyphenol, has also shown to reduce oxidative stress through Nrf2 signaling pathway activation [23]. Our results suggest that the intake of a low dose of polyphenols may be enough to prevent an overproduction of ROS, since the CF diet also offers protection against this increase and provides about 10 times fewer polyphenols than the C10 diet. In line with this, other authors have reported a protective effect of a seven-week dietary intervention, with a 5% cocoa fiber-enriched diet (the same dosage as here) on oxidative stress in adipose tissue of obese rats [29]. The protective effect of cocoa on the excessive ROS production induced by exercise agrees with its effect on the oxidative stress induced by adjuvant arthritis, i.e., a model of chronic inflammation [36]. At the clinical level, a study performed in young elite male football players showed that the consumption of 40 g of dark chocolate (providing 799 µg gallic acid equivalents/mL) per day for 30 days was able to prevent oxidative stress, because the diet prevented the increases in serum soluble NADPH oxidase 2-derived peptide levels, the H_2_O_2_ production, and breakdown activity, as well as the muscle damage observed in control subjects [24]. Other authors also reported the inhibition of the increase in lipid peroxidation induced by exercise after a seven-day cocoa intake [43]. However, they did not observe changes in total plasma antioxidant capacity [43], which agrees with another study showing no changes in the urinary concentration of isoprostanes, which is another oxidative stress biomarker, in rugby players after a seven-day supplementation with a chocolate beverage [48]. 

On the other hand, polyphenols also exert their antioxidant effects by inhibiting enzymes such as xanthine oxidase, NADPH oxidase, and tyrosine and protein kinases involved in the production of free radicals [47], as well as by enhancing the endogenous scavenging antioxidant systems [47]. In previous studies, we found higher thymus superoxide dismutase and catalase activities after a dietary intervention with cocoa in healthy rats [49] and a normalization of both enzymatic activities in the spleen of rats with adjuvant arthritis [36]. Moreover, other authors reported an increase in glutathione reductase activity in mice submitted to downhill running after eight weeks of dietary intervention with several catechins [50]. To our knowledge, there are no available studies assessing the effect of cocoa on the activity of these enzymatic systems in the context of the oxidative stress induced by intensive exercise. Therefore, in further studies, we will assess the effect of a cocoa dietary intervention on the endogenous antioxidant systems after intensive exercise, throughout the study of superoxide dismutase, catalase, glutathione peroxidase, and glutathione reductase activities, as well as quantifying the reduced and oxidized glutathione concentrations. 

Whereas the endogenous production of ROS is essential for maximal force production and training adaptations, an excessive ROS production can affect muscle force by inducing skeletal muscle fatigue and damage, thus leading to a decrease in performance [51]. Here, the rats just ran to exhaustion once at the end of the study, and then, we could not assess the effect of the increase in ROS production on exercise performance. However, we evaluated the potential ergogenic effect of the experimental diets, including cocoa or cocoa fiber. Neither cocoa- nor cocoa fiber-enriched diets modified the exercise performance of the rats. These results agree with most of the human studies considering cocoa polyphenols [28]. With regard to preclinical studies, the available evidence is quite controversial: some studies reported improvements [7,52,53], and others revealed no changes [8] or even a decrease in exercise performance after dietary interventions with cocoa or its isolated polyphenols [54]. Future studies may clarify how the antioxidant properties of cocoa and cocoa fiber, by means of preventing the overproduction of ROS, affect exercise performance in a chronic manner. 

On the other hand, an excessive ROS production can also disrupt immune function and induce an inflammatory status [55], being at least partially responsible for the detrimental effects of intensive exercise on the immune system. A single bout of intensive exercise is able to modify the number and composition of circulating leukocytes [56]. An immediate increase of leukocytes, mainly due to an increase in neutrophils and lymphocytes, followed by a secondary lymphopenia has been extensively described [57]. These alterations are attributed to changes in the hemodynamics, such as vasodilation and increases in heart rate, cardiac output, and blood flow and the action of catecholamines and glucocorticoids on the β2-adrenergic receptors of blood leukocytes in response to exercise [56]. It is well-known that exercise activates the sympathetic nervous system, leading to an increased release of catecholamines and cortisol in a proportional way, i.e., the greater the exercise intensity, the greater the neuroendocrine response [58]. These hormonal changes are normally transient and return to baseline relatively quickly; however, when the intensity of exercise is high and there is not a prior adequate training, the circulating levels of these hormones can be significantly depressed below basal values during the recovery phase [58]. This could explain the decreased plasma levels of cortisol observed in the runner rats 16 h after performing the exhaustion test. These neuroendocrine changes induced by exercise may be responsible for the modifications in the proportions of the circulating granulocytes and lymphocytes. The cocoa diet also decreased the plasma cortisol concentration in the sedentary control group, which agrees with the findings of some clinical studies [59,60]. On the other hand, the impact of acute exercise on the hemoglobin and hematocrit values remains unclear [61]. Most of the available studies, both clinical [61,62,63] and preclinical [64], reported transient decreases in these values due to plasma volume expansion, whereas others found no changes in them after exercise [65,66]. Here, probably due to the timing of sampling (16 h after the exercise bout), the protocol applied did not modify the blood erythrocyte counts, the hemoglobin concentration, or the hematocrit. However, the cocoa intake, especially the diet that contained whole cocoa, induced increases in erythrocyte counts, hemoglobin, and hematocrit. These results are in line with the protective effect of cocoa flavanols and procyanidins on the erythrocyte hemolysis induced by excessive free radicals reported both in humans [67] and rats [68], although the observed increases in erythrocyte variables are also present in the non-exercised animals. Further studies may confirm these results and elucidate the potential mechanisms underlying the higher erythrocyte counts found after cocoa consumption. This could be interesting when considering cocoa or any of its components as a potential ergogenic supplement, because it may enhance muscle oxygenation. 

Acute intensive exercise also impaired blood Ig concentrations. We found that the acute exercise program applied induced a decrease in serum IgG concentration while those of IgA and IgM were not affected. These findings disagree with those reported previously in a chronic rat model of intensive running exercise [26] and with several clinical studies that reported higher levels of IgG in serum immediately after running 90 km [69], although others did not find differences after five weeks [70] and 14 weeks of running training [71]. These controversial results denote the importance of considering the kind of exercise program performed when comparing results among studies. It seems that a chronic moderate-intensity exercise training increases IgG half-life [72], which could explain the higher serum levels of IgG found by others [26,69,70,71], as well as inducing beneficial changes in IgG N-glycosylation that may improve its affinity with the Fc receptor [73]. To our knowledge, the impact of a single bout of intensive exercise without a prior training on serum levels of IgG has not been described before. In fact, most of the studies evaluating the impact of exercise on humoral response focus on salivary IgA for their essential role in mucosal immunity. Anyway, the experimental diets with cocoa and cocoa fiber also decreased serum levels of IgG in the sedentary control rats, and there was not an additional decrease by exercise. Moreover, both the C10 and the CF diets lowered the serum content of IgA in runner rats, and the C10 groups also had lower serum levels of IgM, which agrees with the results of previous studies carried out in non-exercised rats and seems to be attributed to the theobromine content of cocoa [4,12].

## 5. Conclusions

In conclusion, the dietary interventions with cocoa for 25 days were able to avoid the overproduction of ROS induced by a single bout of intensive running exercise, although they were not able to increase the exercise performance or to prevent changes in plasma IgG. Since the cocoa fiber diet also protected against oxidative stress, we can hypothesize that a small content of polyphenols can be enough to counteract this effect. This, together with the beneficial effects of cocoa fiber in the mucosal compartment [11,31,34], such as the prevention of the changes in salivary IgM, the improved cecal short chain fatty acid production, and the decreased secretion of proinflammatory cytokines, makes it a suitable potential supplement to prevent oxidative stress and the microbiota changes induced by an acute intensive exercise. Further research may evaluate the influence of cocoa and cocoa fiber in a more intensive or a longer exercise protocol, which may exacerbate the observed alterations, focusing also on their impact on the endogenous antioxidant systems. It would also be interesting to assess their impact when there is a comorbidity with a disease linked to oxidative stress and their potential protective effect on the induction of an infectious process after exercise, as well as to study possible synergistic combinations with other polyphenols or nutraceuticals.

## Data Availability

Data is contained within the article.
